# Spectrum of Clinical Presentations in Human Immunodeficiency Virus (HIV) Infected Patients with Renal Disease

**DOI:** 10.4314/ajid.v5i2.66509

**Published:** 2011

**Authors:** U H Okafor, E I Unuigbe, F S Wokoma

**Affiliations:** 1 Enugu State University Teaching Hospital Parklane, Enugu; 2 University of Benin Teaching Hospital Benin City; 3 University of Port Harcourt Teaching Hospital Port Harcourt

**Keywords:** HIV, renal impairment, spectrum

## Abstract

HIV infection is a multiorgan disease with the kidney not spared. A variety of renal syndromes with varying clinical presentations has been reported amongst HIV infected patients. This study aims to highlight the spectrum of clinical presentations in HIV infected patients with renal disease. HIV infected patients presenting at University of Benin Teaching Hospital (UBTH) Benin City were the study population. A total of 383 patients were studied. Their biodata, clinical presentations and laboratory investigations including serum urea, creatinine and albumin, urine protein and creatinine were assessed. Their glomerular filtration rate (GFR) and protein urine excretion were calculated using six equations of modification of diet in renal disease (MDRD) and protein: creatinine ratio respectively. Patients were stratified according to their renal functions into normal, mild, moderate and severe renal function impairment. The data was analysed using statistical software program SPSS Vs 15.0. 53.3% of 383 patients screened had renal function impairment, 40.2% mild, 37.7% moderate and 22.2% severe impairment. Mean age was 35.6±8.3, 36.0±9.9 and 36.3±8.3 years for mild, moderate and severe renal function impairment (RFI) respectively. Easy fatigability was the commonest symptoms occurring in 47.5%, 30.0%, 37.5% and 22.5% of control, mild RFI, moderate RFI and severe RFI subjects respectively (p = 0.568). Oliguria, facial and body swelling occurred more in patients with RFI especially in patients with severe renal impairment. The difference is statistically significant (p = 0.046, 0.041, and 0.033 respectively). Pallor was the commonest clinical sign occurring in 32.5%, 50.0%, 35.0% and 62.5% of control and patients with mild, moderate, and severe RFI respectively; the difference was not statistically significant (p = 0.459). Ascites, facial puffiness and pedal oedema were commoner in patients with RFI especially those with severe RFI. The differences were statistically significant. (p = 0.048, 0.019, and 0.008 respectively). In conclusion spectrum of clinical presentations in HIV patients with renal impairment are many but few are specific to these patients.

## Introduction

In 1984, Rao et al reported that renal lesions were found in HIV infected patients. They further described a glomerulopathy, which was characterised by heavy proteinuria, biochemical features of nephrotic syndrome, renal impairment that rapidly progressed to end stage renal disease (Rao et al., 1984). Males and young adults are more commonly affected (Szczech et al., 2004), but nephropathy has been demonstrated in all subsets of HIV infected patients regardless of age, sex, race, and mode of HIV acquisition (Nuermberger, 2007).

Various presentations have been reported in these patients, varying from asymptomatic proteinuria to End Stage Renal Disease (ESRD) with different features of uraemia (Nochy et al., 1993; Winston and Klotman 1996; Seney et al., 1990; Weiner et al., 2002). Urinary abnormalities including oliguria, haematuria, and proteinuria has been reported as presentations in HIV patients with impaired renal function (Moro and Sidhartha 2006; Ijoma, 1996). Extremities oedema and hypertension are not common in these patients but some studies have documented them as clinical presentations of renal impairment in HIV patients especially in patients without HIV associated nephropathy (i.e. non-HIVAN) and acute renal failure (Franceschini et al., 2004; Ruldolph, 2003). Uraemia with its various presentations occur in these patients especially in those with severe renal functional impairment (Iglesias et al., 2006).

There are few studies on the clinical features of HIV patients with renal impairment. This study aims to highlight the spectrum of clinical presentations of HIV infected patients with renal disease.

## Subjects and Method

This is a cross sectional study. Three hundred and eighty three HIV seropositive patients presenting at University of Benin Teaching Hospital (out/inpatients) from 1^st^ January 2007 to 30^th^ June 2007 were randomly selected, and screened for renal function impairment (RFI) using glomerular filtration rate (GFR) which was calculated by using six variable of Modification of Diet in Renal Disease (MDRD) equation. and urine protein excretion using spot urine protein creatinine ratio (PCR). Two hundred and four (53.3%) patients among the total screened had RFI detected by GFR < 60ml/min/1.73m^2^ or PCR ≥ 200. They were stratified into mild (GFR ≥ 60ml/min/1.73m^2^ but PCR ≥ 200), moderate (GFR 30 to 59ml/min/1.73m^2^) and severe (GFR < 30ml/min/1.73ml/min/1.73m^2^) RFI. Eighty two patients (40.2%), 77 patients (37.7%) and 45 patients (22.2%) had mild, moderate and severe RFI respectively. Forty patients were recruited from each stratum by simple random sampling as subjects for the study. Forty patients were also recruited as control by simple random sampling from those patients with normal renal functions detected by GFR >60ml/min/1.73m^2^ and PCR < 200.

Ethical clearance was obtained from the ethical committee of the hospital. The study was explained to the subjects and control. Consent was obtained from each subject and control before the study. The data obtained from subjects and control was documented.

The data was analysed using statistical package SPSS Vs 15.0. The frequency distributions of demographic variables were computed. A one way analysis of variance (ANOVA) was used to test the comparative analysis of clinical presentations as they vary with the severity of RFI. P value < 0.05 was considered significant.

## Results

Two hundred and four (204) of the three hundred and eighty three (383) HIV infected patients screened constituting 53.3% had renal function impairment (RFI). Male constitute 61.7% of patients with renal impairment. The distribution of various severity of RFI is shown in Table 1 below.

**Table 1:** Distribution of severity of RFI

Severity of RFI	Number	Percent
Mild	82	40.2
Moderate	77	37.7
Severe	45	22.2
Total	204	100

### Age distribution

**Figure 1:**
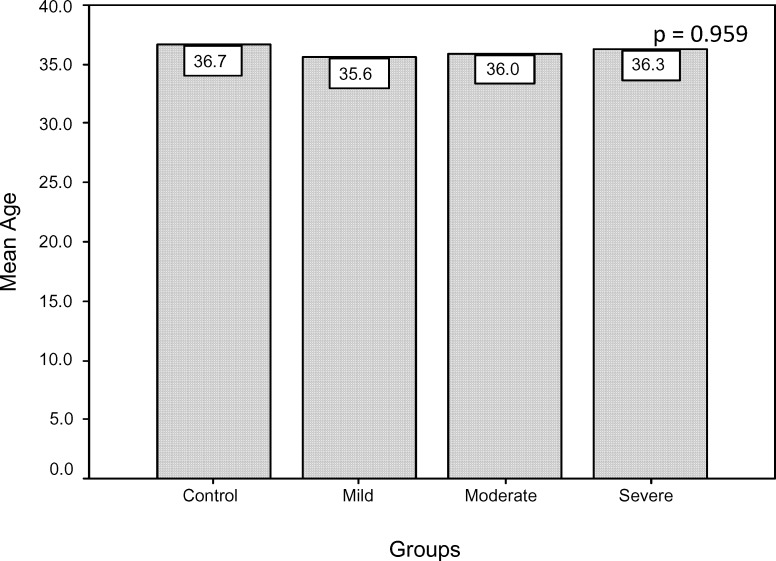
Mean age of subjects with renal impairment and control groups Control − GFR ≥60ml/min/1.73m^2^ & PCR < 200mg/g ; Mild − GFR ≥60ml/min/1.73m^2^ and PCR ≥200mg/g Moderate − GFR 30 − 59ml/min/1.73m^2^; Severe − GFR <30ml/min/1.73m^2^

Figure 1 shows the mean age distribution of patients with various severities of RFI and control. Patients with mild RFI were younger with mean age of 35.6±8.3years but patients with moderate and severe RFI had mean age of 36.0±9.9 and 36.3±8.3 years respectively. There was no statistically significant difference between them (p = 0.95).

### Clinical features

Table 2 shows the major complaints of the subjects and control. Easy fatigability was the commonest symptoms occurring in 19 (47.5%), 12 (30.0%), 15 (37.5%) and 9 (22.5%) of control, mild RFI, moderate RFI and severe RFI subjects respectively. This was not statistically significant (P = 0.568). Nausea and vomiting were commonest in patients with severe RFI occurring in 10(25.0%) and 16(40.0%) of these patients respectively but they were not statistically significant. Oliguria, facial and body swelling also occurred more in patients with RFI especially those with severe renal impairment. The difference was statistically significant (p = 0.046, 0.041, and 0.033 respectively). Pruritus was a commoner symptom in control but the difference was not statistically significant (P = 0.323).

**Table 2:** Symptoms in groups of subjects and control

SYMPTOMS	CONTROL N = 40 (%)	MILD N=40(%)	MODERATE N = 40 (%)	SEVERE N=40 (%)	P VALUE
Easy fatiguability	19 (47.5)	12 (30.0)	15 (37.5)	9 (22.5)	0.568
Weakness of the body	13 (32.5)	9 (22.5)	10 (25.0)	12 (30.0)	0.089
Nausea	9 (22.5)	8 (20.0)	6 (15.0)	10 (25.0)	0.093
Vomiting	5 (12.5)	2 (5.0)	6 (15.0)	16 (40.0)	0.067
Pruritus	12 (30.0)	5 (12.5)	7 (17.5)	2 (5.0)	0.323
Leg swelling	2 (5.0)	3 (7.5)	1 (2.5)	15 (37.5)	0.056
Polyuria	5 (12.5)	3 (7.5)	3 (7.5)	3 (7.5)	0.125
Nocturia	4 (10.0)	4 (10.0)	2 (5.0)	4 (10.0)	0.098
Oliguria	1 (2.5)	1 (2.5)	3 (7.5)	7 (17.5)	0.046*
Facial swelling	1 (2.5)	0 (0.0)	1 (2.5)	8 (20.0)	0.041*
Abdominal swelling	0 (0.0)	2 (5.0)	0 (0.0)	5 (12.5)	0.033*
Loss of consciousness	4 (10.0)	1 (2.5)	0 (0.0)	2 (5.0)	0.254
Haematuria	3 (7.5)	0 (0.0)	1 (2.5)	1 (2.5)	0.098

The major clinical findings in patients with various stages of RFI and control were detailed in Table 3. Pallor was the commonest signs occurring in 13 (32.5%), 20(50.0%), 14(35.0%) and 25(62.5%) of control and patients with mild, moderate, and severe RFI respectively. This was not statistically significant (P= 0.459). Impaired conscious state, fluffy hair, hepatomegaly, splenomegaly, and jaundice were commoner in patients with severe RFI but the differences were not statistically significant. Ascites, facial puffiness and pedal oedema were commoner in patients with RFI especially those with severe RFI. The differences were statistically significant (P = 0.048, 0.019, and 0.008 respectively). The mean systolic and diastolic blood pressure, and body mass index (BMI) were within normal range in both control and subjects.

**Table 3:** signs in groups of subjects

SIGNS	CONTROL N = 40 (%)	MILD N = 40 (%)	MODERATE N = 40 (%)	SEVERE N = 40 (%)	P VALUE
Pallor	13 (32.5)	20 (50.0)	14 (35.0)	25 (62.5)	0.459
Impaired consciousness	0 (0.0)	8 (20.0)	8 (20.0)	14 (35.0)	0.051
Fever	1 (2.5)	5 (12.5)	7 (17.5)	6 (15.0)	0.086
Fluffy hair	3 (7.5)	7 (17.5)	1 (2.5)	7 (17.5)	0.061
Hepatomegaly	1 (2.5)	2 (10.0)	1 (2.5)	8 (20.0)	0.056
Ascites	0 (0.0)	3 (7.5)	0 (0.0)	6 (15.0)	0.048*
Splenomegaly	0 (0.0)	1 (2.5)	0 (0.0)	5 (12.5)	0.056
Facial puffiness	0 (0.0)	0 (0.0)	2(5.0)	3 (7.5)	0.019*
Jaundice	0 (0.0)	0 (0.0)	1 (2.5)	1 (2.5)	0.054
Asterexis	1 (2.5)	0 (0.0)	0 (0.0)	1 (2.5)	0.128
Pedal oedema	0 (0.0)	1(2.5)	2 (5.0)	3 (7.5)	0.008*
Mean systolic BP (mmHg)	115.33±17.17	109.64±17.08	112.11±11.23	118.00±19.34	0.912
Mean diastolic BP (mmHg)	72.33±14.31	74.19±12.21	68.15±10.15	72.52±21.15	0.578
Mean BMI	20.13±4.31	23.9±6.9	22.7±8.8	20.04±4.4	0.652

## Discussion

Renal disorder is a common manifestation in HIV infection. The occurrence rate of 53.3% HIV patients with renal impairment reported in this study is high when compared to other studies (Jones et al., 2007, Ham et al., 2006). However, Agaba in North Central Nigeria reported a prevalence rate of 52%, which compares with this study (Agaba et al., 2003). The mean age of about 36 years and predominant male sex in this study is consistent with other studies (Szczech et al., 2004). Renal diseases presenting as either acute or chronic renal impairment are characterised by varying degrees of clinical features. This depends on the degree of severity of impairment in renal function. In addition, human immunodeficiency virus infection has many and diverse clinical presentations. Thus there is overlap of clinical features attributable to both HIV infection and RFI, such features include easy fatiguability, weakness of the body, nausea, vomiting, pruritus and impaired conscious state (Ijoma, 1996; Onyemelukwe and Musa, 2002; Oyediran and Akinkugbe, 1970). This compares with this study, which showed no statistical significant difference in these symptoms between subjects and control.

Body swelling manifesting as pedal oedema, facial puffiness and occasionally ascites are characteristics features of renal impairment, degree and distribution depends on the type and severity of the renal impairment with facial puffiness as one of the earliest presentations of impaired renal function. Various studies reported body swelling as an uncommon presentation in HIV patients with renal disease, especially in HIV associated nephropathy (HIVAN) (D'Agati and Appel, 1997). This contrast with this study where oedema is a common presentation in HIV infected patients with renal impairment especially those with severe renal function impairment. This may have resulted from added burden of malnutrition prevalent in our environment especially in HIV patients, also hypoalbuminaemia, and vascular abnormality are common in patients with impaired renal function. These promote interstitial fluid exudation and thus the oedema formation. In addition, this study included HIV patients with various causes and type of renal impairment and not just HIVAN. This compares with the studies of Agaba et al and Ijoma that reported oedema as one of the clinical presentations in HIV patients with renal disease.

Urinary symptoms, which include oliguria, polyuria, nocturia, and haematuria, have been reported as common clinical presentations in renal impairment depending on the type/cause of renal disorder (Moro and Sidhartha, 2006; Ijoma, 1996; D'Agati and Appel, 1997). In this study, oliguria was the only urinary symptoms significantly commoner in patients with RFI. This is consistent with study by Ijoma at Enugu Nigeria (Ijoma, 1996). HIV is associated with various other abnormalities independent of renal function that affects frequency and characteristics of urine. These include urinary tract infection, biochemical and renal tubular abnormalities.

Hypertension is a common presentation in both acute and chronic renal impairment in non-HIV patients but has been reported as uncommon in HIV related renal disorder. The mean diastolic and systolic blood pressure in this study is not elevated. The reason for the absence of hypertension is not known.

Anaemia, which present as pallor is a common presentation in HIV infection and renal impairment and this was clearly shown in this study. The causes of anaemia in both clinical conditions are multifactorial. The co-existence of HIV infection and renal impairment worsens the burden of anaemia in these patients in terms of morbidity and mortality (Onyemelukwe and Musa, 2002; Horwich et al., 2002).

Other clinical presentations such as jaundice, hepatomegaly, and splenomegaly may have resulted from other non-renal HIV related or unrelated clinical conditions. Also it is noted that most of the clinical presentations mentioned above were common in patients with severe RFI. This is consistent with observations in non HIV patients whose clinical features vary with severity of RFI. Furthermore severity of RFI has an inverse relationship with the level of CD4 cell count, thus severe RFI may be coexistent with severe immunosupression with antecedent exposure to renal related risk factors like infections, electrolyte abnormality, and drugs (Okafor et al., 2008).

This study acknowledged some limitations which included absence of renal scan, the cross section nature of the study and inability to associate the clinical presentations with various risk factors like low CD4 cell count, drugs, co infections etc contributing to RFI. Thus patients with shrunken kidneys, and transient proteinuria may have been included as patients. However these may not have influenced the result of the study as the patients in this category will have been categorised as normal or mild RFI with unremarkable difference in their clinical presentation.

In conclusion, renal disease is common in HIV infected patients and clinical presentations are many occurring more in severe renal impairment. However, few are specific, thus, use of clinical presentations as the only tool for diagnosis of renal impairment in HIV patients are not reliable. This makes a detailed assessment of renal function in all HIV patients at presentation imperative.
